# Translational research to practice for gastric cancer: molecular classification and outcome prediction based on genomic and proteomic profiling

**DOI:** 10.1186/1479-5876-10-S2-A11

**Published:** 2012-10-17

**Authors:** Youyong Lv, Rui Xing

**Affiliations:** 1 Laboratory of Molecular Oncology, Peking University, School of Oncology, Beijing Cancer Hospital/Institute, Beijing 100142, China

## Background

We have generated genomic and proteomic profiling to approach cellular and molecular mechanism and to discover diagnostic and predictive biomarkers of gastric and colorectal cancer. Based on these gene expression profiles, we explored the characteristics of molecular changes and its potential for clinical significances.

## Materials and methods

As our present data and systemic analyses for gene expression pattern, pathway distribution, gene function category, biosignature and clinical significance shown, we have been able to document the entire gene expression profiles for intestinal- and diffuse-type gastric cancer (GC) and normal appearing tissues (NATs) matched tumors. A group of specific or typical genes were identified as having dramatic changes in tumors and NATs compared with the verified normal samples. Among these genes, at least three gene sets were analyzed using pathway analysis tools and integrated with biological assay data to construct a network for GC carcinogenesis.

## Results

Our data show that these genes are involved in several well-studied signaling pathways associated with development and progression of GC. Our results indicated that alterations of MMP11, MT2A, p42.3, Cyr61 and GKN1 at mRNA and protein level were consistently detected in GC cell lines and primary tumors compared with matched normal tissues. Importantly, serum MMP11 levels were also significantly elevated in GC patients compared with those of the control subjects, and the positive expression was well correlated with metastasis and recurrence in GC patients. As its pilot study, we have generated primary data of its genomic alterations, in combination and comparison with the gene expression profiles. Dramatic correlations have been observed between the gene expression profiles and DNA Copy Number Variations (CNVs), with statistically significant differences between tumors of Stages I-II and III-IV GC. We have also defined a group of specific gene or miRNA alterations, which could be associated with metastasis and recurrence in GC patient.

## Conclusions

Taking together, we have been the first to provide a systematic analysis for comprehensive gene expression profile integrated with microRNA , genomic or proteomic analysis for gastric cancer. Additionally, we have defined a group of genes associated with development and prognosis in gastric cancer.

